# Expression of *GhNAC2* from *G. herbaceum*, improves root growth and imparts tolerance to drought in transgenic cotton and Arabidopsis

**DOI:** 10.1038/srep24978

**Published:** 2016-04-26

**Authors:** Samatha Gunapati, Ram Naresh, Sanjay Ranjan, Deepti Nigam, Aradhana Hans, Praveen C. Verma, Rekha Gadre, Uday V. Pathre, Aniruddha P. Sane, Vidhu A. Sane

**Affiliations:** 1 Plant Gene Expression Lab, CSIR- National Botanical Research Institute, Lucknow-226001, India; 2 Dept. of Plant Physiology, CSIR- National Botanical Research Institute, Lucknow-226001, India; 3 Dept. of Bioinformatics, CSIR- National Botanical Research Institute, Lucknow-226001, India; 4 Plant tissue culture, CSIR- National Botanical Research Institute, Lucknow-226001, India; 5 Dept of Biochemistry, DeviAhilyaBai University, Indore-452001, India

## Abstract

NAC proteins are plant-specific transcription factors that play essential roles in regulating development and responses to abiotic and biotic stresses. We show that over-expression of the cotton *GhNAC2* under the CaMV35S promoter increases root growth in both Arabidopsis and cotton under unstressed conditions. Transgenic Arabidopsis plants also show improved root growth in presence of mannitol and NaCl while transgenic cotton expressing *GhNAC2* show reduced leaf abscission and wilting upon water stress compared to control plants. Transgenic Arabidopsis plants also have larger leaves, higher seed number and size under well watered conditions, reduced transpiration and higher relative leaf water content. Micro-array analysis of transgenic plants over-expressing *GhNAC2* reveals activation of the ABA/JA pathways and a suppression of the ethylene pathway at several levels to reduce expression of ERF6/ERF1/WRKY33/ MPK3/MKK9/ACS6 and their targets. This probably suppresses the ethylene-mediated inhibition of organ expansion, leading to larger leaves, better root growth and higher yields under unstressed conditions. Suppression of the ethylene pathway and activation of the ABA/JA pathways also primes the plant for improved stress tolerance by reduction in transpiration, greater stomatal control and suppression of growth retarding factors.

Plants being sessile in nature have to continuously respond to abiotic and biotic stresses that restrict their growth and yield. Root growth and root system architecture, in particular, are adversely affected by various stresses thereby affecting water/nutrient uptake and aerial plant growth. A robust root system can improve plant growth and aid in stress tolerance. Root growth is under complex hormonal control with most hormones contributing to various aspects of its growth^1^. This control is in turn brought about through regulation by various transcription factors such as those of the ERF/DREB, MYB and the NAC family and by other regulatory proteins^2^.

The NAC family of proteins is a large plant specific family with 100–170 members in most plants^3^ and characterized by a conserved NAC domain at the N-terminal end and a highly divergent transcriptional activator C-terminus^4^. NAC genes, first identified in petunia and Arabidopsis as genes that control shoot apical meristem (SAM) development^5^ were subsequently shown to be involved in other developmental processes such as formation of lateral roots^6^,^7^, secondary wall formation^8^, senescence^9^, petal expansion^10^, fruit ripening/pigmentation^11^ etc. Besides development, these genes are also associated with nutrient remobilization^12^ and resistance to biotic and abiotic stresses^3^,^13^. One of the most prominently studied Arabidopsis NAC genes *RD26* functions in drought stress^14^ while others in rice such as *SNAC1*^15^,^16^ *OsNAC6*^17^, and *OsNAC10*^18^ mediate responses to drought and salt tolerance in rice.

Cotton is a major textiles related cash crop with upland cotton (*Gossypium hirsutum* L.) accounting for >95% of world cotton production. Although India is the second largest producer of cotton after China, it suffers from much lower yields per hectare compared to world average due to growth on rainfed soils and uneven and erratic monsoon conditions in India. Varieties with robust root systems and better abiotic stress tolerances need to be developed through breeding or genetic manipulation. In this paper, we show that expression of *GhNAC2*, isolated from the relatively drought tolerant *G.herbaceum*, leads to much improved root growth under well watered conditions both in transgenic Arabidopsis and cotton and primes the plant for improved drought tolerance through modulation of the ABA/JA and ethylene pathways.

## Results

The *GhNAC2* gene (Acc no EU706339^19^) was obtained as a root expressed gene using *G. herbaceum* root cDNA as template. The NAC domain, present towards the N-terminal end, possesses all the five motifs described in NAC proteins. In addition, it also contained a NARD like sequence from amino acids 104–132 (Fig. S1). This motif functions as a repressor when fused to VP16 in plant protoplast assays and has been reported in other repressor type NAC members such as GmNAC20, GmNAC11, AtNAC2, NST1, ATAF1, RD26 and SNAC^7^,^10^,^20^.

### Expression of *GhNAC2*

**Figure 1:**
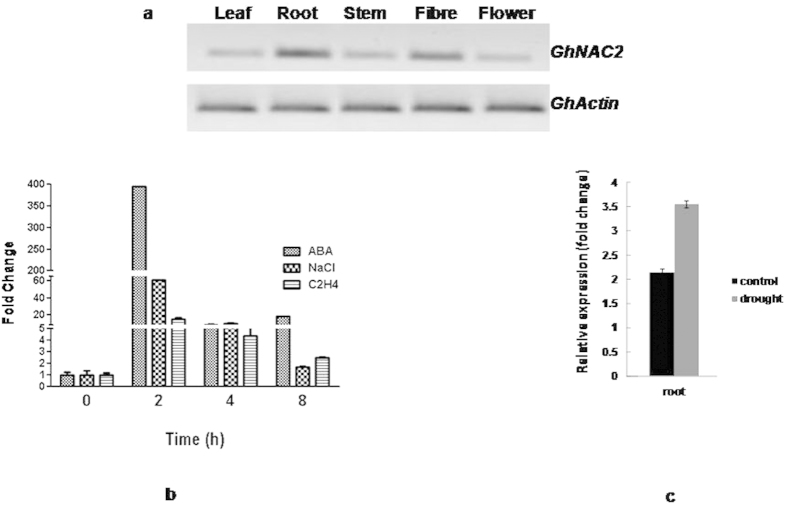
Transcript accumulation of *GhNAC2* in different tissues and under various stress conditions in cotton. (**a**) mRNA abundance of *GhNAC2* in vegetative tissues by semi-quantitative RT-PCR. Actin was used for normalization. (**b**) Real time quantification of *GhNAC2* transcript abundance in cotton leaves after ABA, ethylene and NaCl treatments. Actin was used for normalization. The expression of the gene in control (untreated samples) was taken as 1 and relative expression in all samples (in subsequent time points) plotted against it. Vertical bars indicated ± Sd of three technical replicates of pooled treated samples. (**c**) Real time quantification of *GhNAC2* transcript abundance in roots under control and drought conditions. Expression was studied in plants (containing 4–5 leaves) exposed to water stress for 30 days.

To understand the regulation of *GhNAC2* in different cotton tissues, its transcript accumulation patterns were determined by semi-quantitative PCR. The analysis revealed expression of *GhNAC2* in leaf, root, stem, flower and fibre (30 days post anthesis) with relatively higher transcript accumulation in root and fibre (Fig. 1a). Since NAC genes are also involved in and are regulated by abiotic stresses and hormones governing these, the expression of *GhNAC2* was studied in response to stress related hormones like ABA and ethylene and under salinity stress (using NaCl) by qRT-PCR. As shown in Fig. 1b, *GhNAC2* was strongly induced by ABA (~400 folds) within 2 h compared to water treated leaves followed by a gradual decrease from 4–8 hours. Salt and ethylene treatments also induced peak increases of about 60 and 20 folds respectively within 2 h followed by a decrease. The expression of *GhNAC2* also increased in drought exposed roots by approximately 1.5 folds compared to control roots (Fig. 1c).

### Subcellular localization of GhNAC2

**Figure 2:**
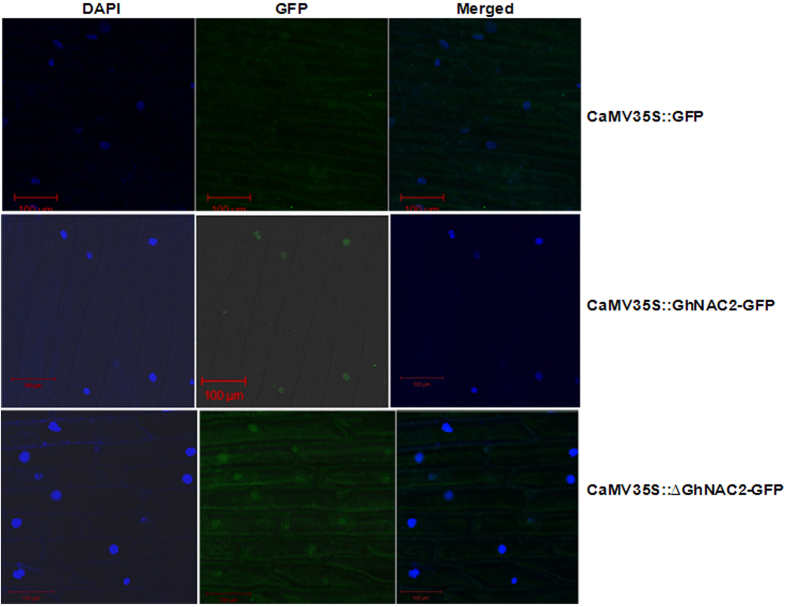
Subcellular localization of GhNAC2-GFP fusion protein. The fusion proteins CaMV35S::GFP, CaMV35S:GhNAC2-GFP and CaMV35S:ΔGhNAC2-GFP were transiently expressed in onion epidermal cells by particle bombardment (as described in the methods section) and visualized under a confocal microscope.

To find out the sub-cellular localization of GhNAC2 protein, a GhNAC2-GFP translational fusion construct under CaMV35S promoter was introduced into onion epidermal cells by particle bombardment. The GhNAC2-GFP fusion protein was localized to the nucleus, unlike the control CaMV35S::GFP where florescence was present throughout the cell (Fig. 2). Florescence in onion cells bombarded with CaMV35S:ΔNAC-GFP construct (that lacked most of the NAC domain) was distributed throughout the cell besides the nucleus. These experiments indicated that the GhNAC2 protein was localized in the nucleus and that the nuclear localization signal was present within the NAC domain.

### *GhNAC2* expressing Arabidopsis show improved root growth

**Figure 3:**
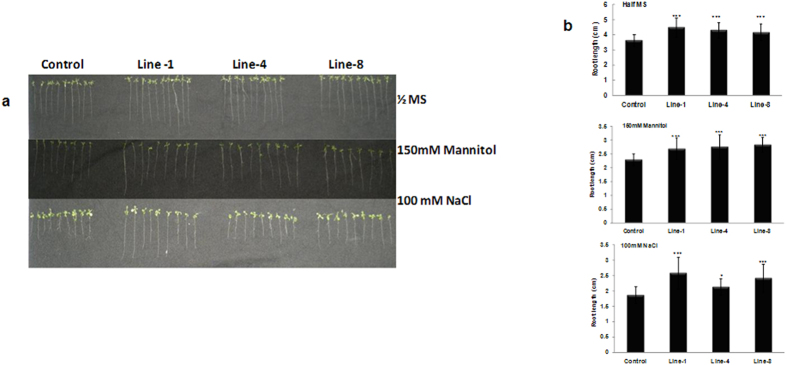
(**a**) Comparative root growth in control and transgenic *GhNAC2* expressing plants after various treatments. Seeds of independent transgenic lines (L-1, L-4 and L-8) were germinated on ½ MS supplemented with 150 mM mannitol/100 mM NaCl and root length measured after 15 days. (**b**) Graphical representation of the root length measured in control and transgenic *GhNAC2* expressing plants shown in Fig. 3a. Error bars indicate ± Sd; n = 10. *P < 0.05; ***P < 0.001.

In order to get an insight into the function of *GhNAC2*, homozygous progeny of three independent transgenic *Arabidopsis* lines (Lines 1, 4 and 8) constitutively expressing the full length *GhNAC2* under the CaMV35S promoter were chosen for study (Fig. S2). As shown in Fig. 3a,b, plants of all transgenic lines showed longer roots than the control when grown on ½ MS medium. Compared to a root length of 3.66 ± 0.4 cm in controls, transgenic lines had root lengths of 4.84 ± 0.5 cm (Line 1), 4.5 ± 0.5 cm (Line 4) and 4.4 ± 0.3 cm (Line 8) representing an increase of about 20–32% over the control even in absence of any stress. Comparative differences in root growth were also observed under various stress conditions (150 mM mannitol and 100 mM NaCl). Upon treatment with mannitol, control plants showed a root length of 2.24 ± 0.21 cm. The transgenic lines had root lengths of 2.65 ± 0.44 cm (Line 1), 2.7 ± 0.5 cm (Line 4) and 2.8 ± 0.23 cm (Line 8) representing an increase of about 18–25% over the control (Fig. 3b). Transgenic *GhNAC2* lines also showed a 15–31% increase in root length upon salinity stress (100 mM NaCl) with lengths of 2.54 ± 0.46 cm (Line 1), 2.38 ± 0.18 cm (Line 4) and 2.76 ± 0.38 cm (Line 8) compared to the control (2.1 ± 0.5 cm). These results suggested that *GhNAC2* expression improved root growth in transgenic plants under unstressed and water/osmotic stress conditions in the initial stages of seedling development.

### Transgenic *GhNAC2* lines show better vegetative growth and productivity

**Figure 4:**
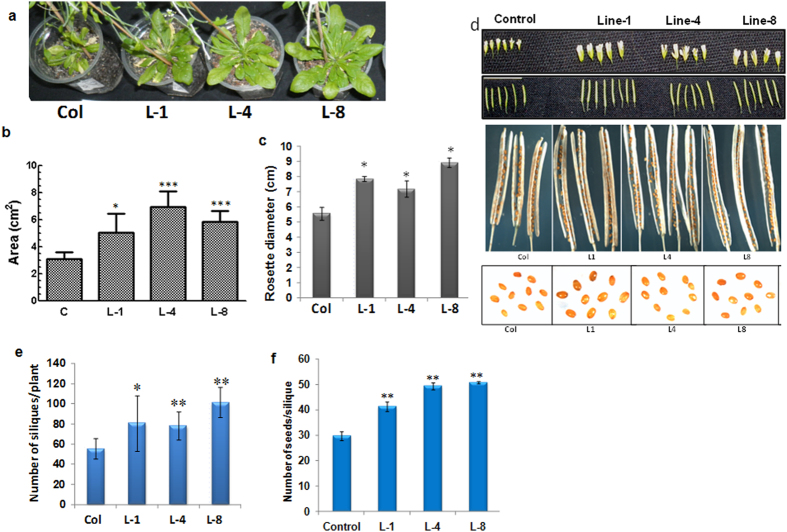
Comparative growth of control and *GhNAC2* expressing transgenic Arabidopsis plants. (**a**) Leaf and rosette size variation. (**b**) Graphical representation of the leaf surface area of control (Col) and transgenic lines (n = 10). (**c**) Graphical representation of the rosette diameter of control (Col) and transgenic lines (n = 5). (**d**) Flower size variation, silique size variation, seed number variation, seed size variation. (**e**) Graphical representation of number of siliques/plant (n = 5). (**f**) Graphical representation of number of seeds/silique (n = 10). *P < 0.05, **P < 0.01.

Besides improved root growth, all *GhNAC2* expressing transgenic lines showed better growth under normal conditions with larger leaves and a greater vegetative growth (Fig. 4a). Surprisingly, although the timing of appearance of floral buds did not differ in transgenic plants compared to controls, bolting of the inflorescence was delayed by about two days in transgenic plants (Fig. S3). Leaf area of transgenic plants increased by 65–140% (Fig. 4b) while rosette diameter increased by about 35–63% (Fig. 4c). Transgenic lines also had larger flowers, longer siliques, greater seed number and greater seed size (Fig. 4d) under normal growth conditions compared to control. The improved growth resulted in a net increase in yield in transgenic lines with a 45–80% increase in silique number per plant (Fig. 4e) and 33–66% increase in number of seeds/silique (Fig. 4f).

### Expression of *GhNAC2* confers drought tolerance in Arabidopsis

**Figure 5:**
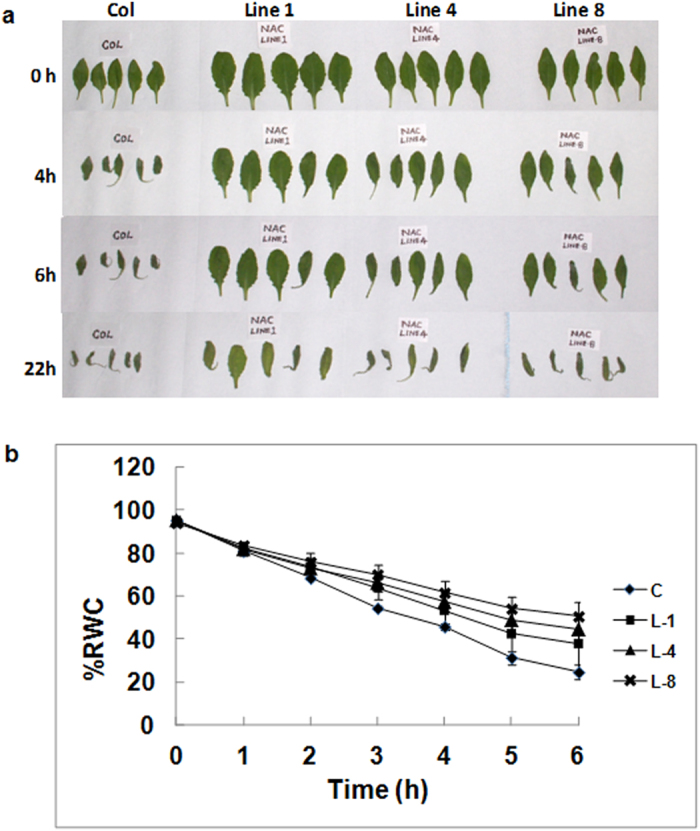
(**a**) Comparative leaf drying analysis in excised leaves (seventh leaf from bottom) of transgenic *GhNAC2* expressing (lines L-1, L-4 and L-8) and control Arabidopsis plants over a 24 hour period. Experiments were performed in triplicates in independent lines (5 plants/line/set). (**b**) Graphical representation of changes in relative water content of excised leaves of transgenic *GhNAC2* (Lines L-1, L-4 and L-8) Arabidopsis lines with time. Rosette leaves (seventh leaf from bottom; 5 plants/line/set) were detached and leaf weight measured at indicated time points. Each data point represents the mean of triplicate experiments.

Since *GhNAC2* transgenic plants showed improved root growth even under water stress conditions, we tested whether transgenic leaves had greater tolerance to water stress. A comparative time course study of the relative water content of detached leaves of control and transgenic *GhNAC2* expressing lines revealed a marked difference in the time taken to wilt and shrivel between control and transgenic lines (Fig. 5a). Leaves of control plants started showing wilting symptoms, petiole upturning and leaf curling within 2 h of detachment. By 6 hours, the detached leaves had wilted and started shrivelling. In contrast, excised *GhNAC2* leaves retained their normal shape with only traces of wilting at 2 and 6 h of detachment. By 24 hours both control and transgenic leaves showed shrivelling of leaves. A graph of percent relative water content versus time showed a clear reduction in the rate at which water was lost in the transgenic leaves compared to the control (Fig. 5b). At 1.5 h, control leaves had lost about 28–30% water while by 3 h they lost about 45% water. In contrast, transgenic lines took twice as much time for the same water loss with a 25–32% loss in water in 3 h and 45–55% loss in 6 h (by which time detached control leaves retained only about 25% water). Transgenic *GhΔNAC2* expressing plants lacking the NAC domain started showing wilting symptoms much earlier although slightly later than control leaves (Fig. S4).

In view of the reduced water loss in transgenic plants we next studied whether transgenic lines had a better control over transpiration and water use efficiency through physiological studies. As shown in Table 1, transpiration (E) decreased from 3.92 ± 0.90 mmol H_2_O m^−2 ^s^−1^ in controls to 2.01 ± 0.48 mmol H_2_O m^−2^ s^−1^ in L-1, 1.98 ± 0.21 mmol H_2_O m^−2^ s^−1^ in L-4 and 2.51 ± 0.17 mmol H_2_O m^−2^ s^−1^ in L-8 transgenic lines. This represented a decrease of about 50% in lines L-1 and L-4 and 37% in L-8 compared to controls indicating reduced water loss in transgenic lines. Stomatal conductance (Gs) was also reduced in all three transgenic lines with values of 683 ± 229 mmol H_2_O m^−2^ s^−1^ in controls compared to values of 330 ± 71 to 565 ± 119 mmol H_2_O m^−2^ s^−1^ in lines L-1, L-4 and L-8. This reduction was associated with a 38–50% decrease in photosynthetic rates in transgenic lines as against control with values of 5.63 ± 1.28 μmol CO_2 _m^−2^ s^−1^, 4.53 ± 0.32 μmol CO_2 _m^−2^ s^−1^ and 5.40 ± 0.35 μmol CO_2 _m^−2^ s^−1^ in lines L-1, L-4 and L-8 respectively compared to 9.07 ± 1.98 μmol CO_2 _m^−2^ s^−1^ in controls. However, the intrinsic water use efficiency (WUE) calculated as A/E ratio did not show any significant change between control and transgenic lines (Table 1). No change was observed in the Fv/Fm ratios (which were ~0.83) in transgenic lines suggesting that the function of reaction centres was not altered (data not shown).

### Microarray analysis of transgenic *GhNAC2* expressing lines

In view of the improved growth in the transgenic lines under unstressed conditions, a comparative expression profile of CaMV35S:GhNAC2 Arabidopsis plants (Line 1) with control was carried out by microarray analysis using Affymetrix ATH whole genome array. Using stringent filtering options, 137 up-regulated and 371 down-regulated genes affecting hormone signalling, carbohydrate metabolism, energy, signal transduction, responses to biotic/abiotic stresses etc. were identified in the *GhNAC2* expressing lines. The most pronounced changes were observed in the pathways that govern ethylene and ABA and JA signaling. Prominent genes involved in ethylene biosynthesis (*ACS6, MPK3, MKK9* and *WRKY33*) and response (*ERF1, ERF2, ERF6, ERF11* and others) were down-regulated compared to control (Table 2). Simultaneously, transcript levels of *EBF2* (involved in proteasomal degradation of EIN3) were up-regulated in transgenic lines. In contrast, the ABA pathway appeared to be up-regulated as seen from the higher expression of *AtPYL8*, (encoding an ABA receptor), and reduced expression of *AHG3* (a negative regulator encoding PP2C) and *CYP707A3*, encoding an ABA 8′ hydroxylase that irreversibly inactivates ABA, in transgenic lines. Four WRKY members, *AtWRKY18, WRKY33, AtWRKY40* and *AtWRKY70* that act as negative regulators of ABA action were also down-regulated suggesting activation of ABA responses by *GhNAC2* at several levels. Other genes that affect ABA and stress responses such as *CIPK11, CIPK14, ERD9* (a glutathione S transferase), *MYB44* (an activator of WRKY70), *STZ, RD26, RD29A* were also down-regulated. Interestingly some of these, although positive regulators of ABA and drought stress signaling, actually suppress growth when over-expressed. The JA pathway also appeared to be affected. At least 6 members of JAZ family of repressors besides *CYP91A* (involved in catabolism of JA) were considerably down-regulated indicating possible activation of jasmonate responses in transgenic lines. Intriguingly, genes of jasmonic acid biosynthesis such as lipoxygenase (*LOX3, LOX4*), allene oxide synthase (*AOS*), allene oxide cyclase (*AOC3* and *ERD12*) and response (*MYC2*) were also down regulated (Table 2). Genes related to the glucosinolate pathway and sulfur metabolisms were also affected.

### Transgenic *GhNAC2* expressing cotton show improved root growth

**Figure 6:**
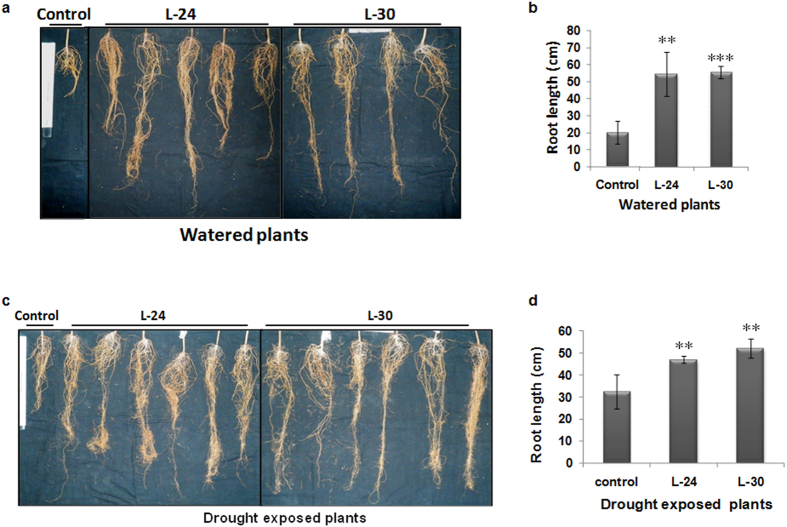
Comparative root growths of control and transgenic cotton plants expressing *GhNAC2* under the CaMV35S promoter in well-watered and water-stressed conditions. Control (Coker) and transgenic *GhNAC2* expressing cotton plants (lines L-24 and L-30) were grown in Neopit under well-watered conditions for two months and then subjected to 15 day water stress (water withheld for 15 days) followed by re-watering and root length measured after maturity (5 months 10 days). The control lot (Coker and transgenic lines) was watered continuously during this period (**a**) Root length in well watered control and transgenic *GhNAC2* expressing lines. (**b**) Graphical representation of root length of plants in Fig. 6a (n = 6). (**c**) Root length in 15 day drought exposed and re-watered control and transgenic *GhNAC2* expressing lines. (**d**) Graphical representation of root length of plants in Fig. 6c (n = 6). **P < 0.01; ***P < 0.001.

We also investigated the effects of *GhNAC2* expression under the CaMV35S promoter in cotton (Coker 310). Interestingly, a marked increase in root length (as seen in transgenic Arabidopsis lines) was also seen in all transgenic *GhNAC2* expressing plants both under well watered and water stressed conditions. Under unstressed conditions, plants of transgenic lines of L-24 and L-30 showed a 2.5–2.75 fold increase in root length over control roots (Fig. 6a,b) while under water stressed conditions plants of transgenic lines of L-24 and L-30 showed a 1.5- 1.7 folds over control roots (Fig. 6c,d).

**Figure 7:**
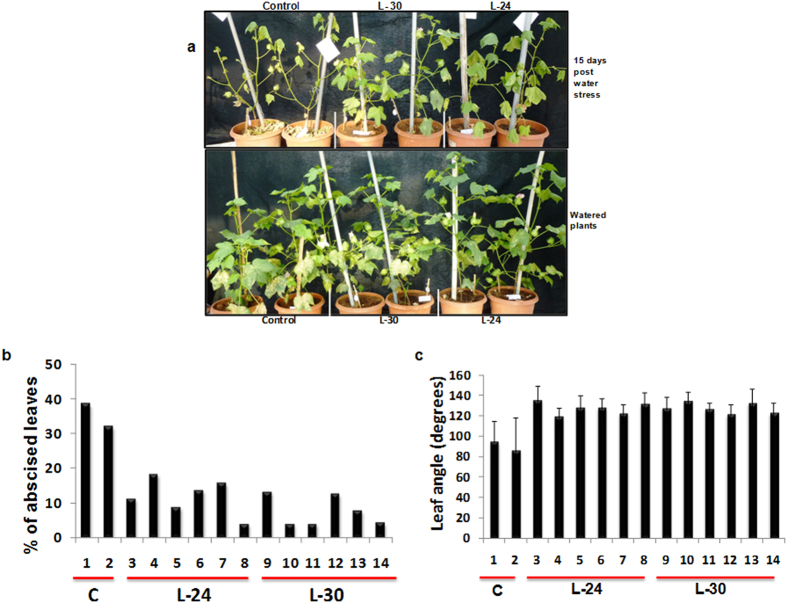
Comparative plant phenotypes of control and transgenic *GhNAC2* expressing cotton plants under water-stressed and well-watered conditions. (**a**) Comparative plant growth phenotypes of control and transgenic *GhNAC2* expressing cotton plants described in Fig. 6 under water-stressed (upper panel) and well-watered conditions (lower panel). (**b**) Graphical representation of percent abscission of leaves in control and progeny of various transgenic *GhNAC2* expressing cotton plants (Lines L-24 and L-30) after water stress (n = 6). (**c**) Graphical representation of leaf angle of leaves remaining in control and progeny of various transgenic *GhNAC2* expressing cotton plants (Lines L-24 and L-30) after water stress. Leaf angle (10 leaves/plant) was measured as the angle between the petiole and leaf lamina (n = 6).

Leaf abscission and wilting, which are common drought symptoms in cotton, were found to be reduced in transgenic plants subjected to 15 days water stress (Fig. 7a). While leaves of control cotton plants showed an average of 35% leaf abscission post 15 day water stress, the average leaf fall in plants of transgenic lines L-24 and L-30 was only 11.8% and 7.4% respectively (Fig. 7b). Of the leaves that remained, there was a greater degree of wilting in water stressed control plants compared to transgenic lines. Leaf drooping measured as the angle between the leaf laminal plane and the petiole was 90.25° ± 8.2 in control leaves compared to 120–137° for lines L-24 and L-30 after 15 days of withholding water, indicating reduced wilting in transgenic plants (Fig. 7c).

## Discussion

NAC transcription factors constitute a large plant-specific family that are involved in many regulatory and developmental processes as well as stress responses in several plants. Nevertheless, functionality studies of individual members have been restricted mostly to Arabidopsis with very few studies in other plants^3^. In cotton, about 73 NAC domain proteins belonging to four subfamilies namely, ATAF, AtNAC3, NAM and NAP were identified in three different studies in *G. hirsutum*^19^,^21^,^22^ while about 145 NAC genes were identified in *G. raimondii*^23^. One of these, *GhNAC2*, was also identified in our screens during a search for root related NAC cDNAs in cotton. The gene was reported as not being expressed in *G. hirsutum* roots^19^ which is a drought susceptible variety, as compared to *G. herbaceum* used in our case.

Constitutive *GhNAC2* expression markedly improves root growth (both primary and lateral) under well watered conditions both in transgenic Arabidopsis as well as transgenic cotton with an increase of almost 20–30% in length in Arabidopsis and a 150–175% in cotton (Figs 3 and 6) suggesting a role in regulation of root length and root growth. Several previous NAC genes have been shown to be involved in root development. Some of these such as *AtNAC1, AtNAC2* and *GmNAC20* function specifically to improve lateral root growth in transgenic lines^6^,^7^,^24^ although GhNAC2 also improved primary root length, especially in cotton. Microarray analysis of transgenic *GhNAC2* expressing plants shows differential regulation of over 500 genes of which thrice as many (371) are suppressed as are up-regulated (137). This points to a repressor like function for GhNAC2 and is in tune with the presence of the NARD repressor motif in GhNAC2 just as described for genes such as *RD26, GmNAC20* and *RhNAC*^10^,^20^. The most prominent effect is the suppression of the ethylene pathway at various levels and includes down-regulation of *ACS6* (involved in stress ethylene production), *WRKY33, MPK3* and *MKK9* (which activate the *ACS6* gene and protein^25^), *ERF1, ERF2, ERF6* and other ERFs (that mediate ethylene responses), and an increase in EBF2 (which reduces EIN3 levels) (Table 2). Ethylene inhibits cell elongation and suppresses organ growth and expansion in roots, leaves, petals etc.^10^,^26^ and mutants over-expressing ethylene biosynthetic/response genes show much reduced growth^27^. Thus, the *GhNAC2* mediated inhibition of the ethylene pathway as seen in transgenic lines, along with activation the GA pathway (by suppression of *GA2OX2* involved in GA metabolism), would explain the increase in root length, rosette diameter and the 1.6–2.4 fold increase in leaf area. The large increase in leaf surface area leads to greater net photosynthesis that translates into greater seed size and number and more than compensates for the reduced photosynthesis rates during early stages.

Simultaneously, the suppression of ethylene pathway and activation of the ABA pathway primes the plant for greater tolerance to abiotic stresses. This is evident from improved root growth under mannitol and NaCl treatments in Arabidopsis, a marked reduction in water loss upon leaf detachment, a delay in leaf wilting and the higher leaf relative water content in transgenic leaves. Indeed, transgenic leaves take almost twice as long to lose the same amount of water as the controls – an effect that can be explained by the two fold reduction in transpiration compared to controls (Table 1). In this respect, *GhNAC2* could also be considered as a stress associated NAC (SNAC)^17^ and, like many other SNACs, its expression is under a strong transcriptional regulation by ABA, ethylene and NaCl (Fig. 1A). Several SNACs such as *RD26, ANAC096, OsNAC1, OsNAC6, GmNAC6* and *GmNAC20* have been shown to differentially regulate drought and salt responses in Arabidopsis and rice (reviewed by Nakashima *et al.*)^13^. *RD26* expression improves drought responses although normal growth (in absence of stress) is affected^14^. In rice, *SNAC1* expression reduced transpiration through stomatal closure without affecting photosynthetic rates thereby maintaining yields^15^ and had no adverse effects on root growth. Its expression in cotton improved root growth^16^. Root specific expression of *OsNAC10* improved drought tolerance and yield and generated thicker roots^18^ unlike when expressed in the whole plant. Thus different NAC genes modulate the expression of different sets of genes in response to drought to bring about tolerance in different ways.

*GhNAC2* mediates its responses through combinatorial effects on different hormonal pathways that lead to increased ABA (and possibly JA) responses and reduced ethylene responses. Expression of the ABA receptor, *AtPYL8*, was up-regulated while those encoding negative regulators of ABA namely *AHG3, CYP707A3*, *AtWRKY18*, *AtWRKY40* and *AtWRKY70* were down-regulated. Since *wrky70* mutants show improved water retention and enhanced stomatal closure^28^, a reduction in *AtWRKY70* transcript levels would improve water retention as actually observed in our studies. *MYB44*, a transcriptional activator of *WRKY70* retards growth through its expression^29^ and is also reduced in transgenic *GhNAC2* lines. Similarly, WRKY18, WRKY40 and WRKY60 function in a complex that represses ABA responses^30^. Their suppression (Table 2) would therefore enhance ABA responses. Another down-regulated gene, *ERD9*, encoding a glutathione S transferase, functions as a negative regulator of drought responses with mutants showing higher ABA levels, lower transpiration and improved primary and secondary root growth^31^ - phenotypes that are seen in *GhNAC2* lines. Other negative regulators of ABA responses such as *CIPK11* and *CIPK14* were also down-regulated. Interestingly, several stress activated genes like *RD26, RD29B, NAC3 (ANAC055), WRKY33, STZ, MYB44*, and a CCCH type Zn finger gene (At3g55980) were also down-regulated in transgenic *GhNAC2* expressing plants. Expression of some these genes leads to growth retardation^14^ indicating that these may activate more drastic survival responses that reduce growth as against a drought avoidance response that may activate water retention processes while maintaining growth. The JA pathway was also affected with genes encoding JA biosynthesis enzymes such as *LOX3, LOX4*, *AOC3* and *AOS* as well as those encoding JAZ proteins (inhibitors of JA action) being strongly down-regulated. JAZ inhibitors prevent JA transcription factors from functioning in absence of JA but are degraded by JA mediated proteasomal degradation by the SCF^COI^ complex^32^. Thus, even with reduced levels of JA, the lack of JAZ proteins would lead to a net activation of JA responses. JA has recently been shown to induce stomatal closure and this induction requires ABA action^33^. In tomato, JERF1 activates ABA biosynthesis in drought^34^. These studies indicate that the concerted increase in ABA and possibly JA responses may prime the plant in mounting a more effective response to drought^35^.

The ethylene pathway is also activated in drought. In cotton, water stress specifically enhances ethylene mediated leaf abscission and senescence^36^. Thus, the suppression of the ethylene pathway through GhNAC2 as seen in Arabidopsis, might explain the reduction in water stress associated leaf abscission and senescence that we observe in transgenic cotton. These observations are similar to those of Manavella *et al.*^37^ who showed that over-expression of *Hahb-4* from sunflower led to strong tolerance to water stress and down-regulation of genes related to ethylene biosynthesis (ACO and SAM synthetase) and ethylene signalling (ERF2 and ERF5). Ethylene has also recently been shown to actively suppress cell division and elongation, thereby reduce plant growth in response to mild osmotic stress^38^. These responses were mediated by ERF5 and ERF6 as master regulators which were rapidly phosphorylated and activated by MPK3 and MPK6 leading to (1) activation of stress response genes like *WRKY33, STZ* and *MYB51* and (2) suppression of cell division/elongation through reduced GA availability by activation of *GA2OX6*^39^. Interestingly, *GhNAC2* expression inhibits both these responses. It not only suppresses *ERF6* transcription but also *MPK3, MKK9, ACS6* and other positive regulators of ethylene like *ERF1* and *ERF2* besides suppressing *GA2OX2*. It simultaneously reduces transcript accumulation of *AtWRKY33* and *STZ/ZAT10*. Collectively, these changes by GhNAC2 would suppress the growth inhibition by ethylene and increase organ size through cell elongation under unstressed conditions. At the same time its action would promote greater transpirational control (without affecting water use efficiency) through regulation of genes affecting stomatal closure and prevent activation of stronger stress responsive genes such as *RD26, MYB44, STZ/ZAT10* that reduce growth under stressed conditions. Two recent studies have made similar observations in cotton regarding stress responsive pathway activation. Padmalatha *et al.*^39^ showed that drought activated several stress response genes leading to inhibition of fibre elongation^40^. Ranjan *et al.*^41^ also observed differences in expression of stress associated genes between drought sensitive and tolerant varieties of *G. herbaceum*^41^. Most of these genes, activated by severe stress, are actually down regulated in our studies upon over-expression of *GhNAC2* thus allowing the plant to combat drought without seriously affecting growth. In another study in the *immature* mutant of cotton, which has reduced fibre thickness and reduced fibre elongation in early stages, transcriptomic analysis revealed deregulated expression of genes associated with severe stress but reduced *GhNAC2* expression in fibre^42^. The direct association between *GhNAC2* expression and cell elongation observed in this study and the inverse correlation with genes associated with severe stress that reduce growth is also seen in our studies. These studies and recent observations in our lab seem to indicate a role for *GhNAC2* in cell elongation in both root and fibre (where *GhNAC2* is highly expressed; Fig. 1) through hormonal response control.

In conclusion, we show that *GhNAC2* action improves root growth in both transgenic Arabidopsis and cotton under unstressed conditions while its action primes the plant for a better stress response through simultaneous modulation of the ABA/JA and ethylene signal pathways in a way that maintains growth under stressed conditions (through improved water retention) in Arabidopsis as well as cotton. This makes *GhNAC2* an attractive candidate for genetic manipulation for drought stress tolerance in cotton and other commercially important plants.

## Methods

### Plant material and treatments

Cotton plants (*G. herbaceum*, *var*. Vagad) were grown in vermiculite at 30–32 °C in a glasshouse and supplemented with Hoagland solution twice a week. For drought stress treatments, water was withheld for 30 days in plants at the 4–5 leaf stage. Plants were sacrificed after 1 month of water stress (16.5% soil moisture content as measured using a wet sensor moisture meter, ∆T WET Sensor Kit from Delta-T Devices, Cambridge, England) and root samples frozen in liquid nitrogen and kept at −70 °C till further use. For ABA and NaCl treatments, one month old plants grown in vermiculite were carefully removed and placed in a beaker containing either water or 100 mM NaCl or 100 μM ABA. For ethylene treatment, plants were exposed to 10 μl/L ethylene in an enclosed chamber for 8 h. Leaf samples were collected at 0 h, 2 h, 4 h and 8 h time intervals, frozen and stored at −70 °C until further use.

### RNA isolation, cDNA preparation and gene isolation

RNA was isolated from and frozen cotton roots (1–2 mg) using Sigma RNA isolation kit (Sigma Aldrich) and first strand cDNA prepared using the Revertaid MuMLV Reverse transcriptase (Fermentas) and 3′adapter primer 3′AP (Invitrogen). Two sets of degenerate primers (NACF1, NACF2, NACR1 and NACR2; supplementary Table S1) were designed from the conserved regions of NAC DNA sequences and used for amplification of a partial 257 bp fragment using drought exposed cotton root cDNA as template. Amplified fragment was cloned and based on its sequence, gene specific primers NACF3, NACF4 were designed and used in combination with 3′AP to amplify a 1225 bp fragment that included the 3′ end. This showed 100% identity to the EST clone later designated as *GhNAC2* by Meng *et al.*^19^. Specific primers F0 and R0 were then synthesized to obtain the complete ORF.

### Transcript analysis of *GhNAC2* by semi-quantitative PCR and real time PCR

Total RNA was extracted from different tissues and control and drought exposed roots and analysed with the NACF3/NACR3 primer combination by semi-quantitative and qRT-PCR with actin as internal control (supplementary Table S1). qRT-PCR, in technical triplicates of pooled treated samples, was performed using SYBR GREEN PCR Master Mix (PE-Applied Biosystems, CA, USA) on an ABI PRISM 7000 sequence-detection system. Relative fold differences were calculated based on the comparative Ct method using actin as an internal standard for normalization.

### Nuclear localization of GhNAC2

For study of the sub-cellular localization of GhNAC2, the GhNACF0-GhNACRP fragment was cloned in vector pBIGFP at *BamHI* site in fusion with GFP gene at N-terminal end under the control of CaMV35S promoter. Onion epidermal cells were bombarded with gold particles coated with 2 μg of recombinant plasmid or pBIGFP using a Bio-Rad PDS1000/He Biolistic particle delivery system (rupture disk of 900 psi). For localization of the NAC-GFP protein, sections were observed under a LSM510 META confocal microscope (Zeiss) using a neon laser (excitation at 395 nm, emission at 509 nm). For visualization of nuclei, the tissue was stained with DAPI (4,6-diamidino-2 phenylindole dihydrochloride, 300 nM in 1XPBS pH 7.2) for 5 min and observed using diode 405 laser (excitation at 350–365 nm, emission in the range of 435–485 nm). For image merging, processing and assembly, the inbuilt software was used.

### Plasmid construction and transformation of Arabidopsis and cotton

For functional analysis in Arabidopsis, the *GhNAC2* gene and *Gh∆NAC2* version (lacking the N-terminal NAC domain) were cloned under the CaMV35S promoter in pBI121. The recombinant clones were introduced into wild-type *A. thaliana* (ecotype Columbia) by the Agrobacterium based floral dip method^43^. Seeds were selected on kanamycin, screened for presence of transgene and subsequently grown to homozygous T3 generation. Three independent homozygous T3 lines expressing *GhNAC2* (L-1, L-4 and L-8; 3–5 plants each) were used for functional studies that included analysis of root length, leaf size, rosette diameter, silique length, seed number/silique and seed size.

### Cotton transformation and drought related experiments in transgenic lines

Besides Arabidopsis, the CaMV35S:GhNAC2 construct was also introduced into cotton (*G. hirsutum*, Coker 310 variety) by transformation of the shoot apical meristem as described by Kumar *et al.*^44^. Transgenic plants were selected on kanamycin on the basis of presence of root-laterals in selection medium (confirmed later by PCR) and then transferred to pots containing a mixture of sandy loam, sand, vermiculite and peat moss in 2:1:1:1 ratio. The plants were covered with polythene bags to maintain high humidity for 14–18 days of hardening in a glasshouse at 28 ± 2 °C, 60 μmol m^−2^ s^−1^ light intensity and 16 h photoperiod and later transferred to a net house. T0 seeds from positive lines were grown to T1 and T2 generation (screened at every stage for transgene by PCR). Plants in the T2 generation from two independent lines, L-24 and L-30, were used for analysis.

### Root length measurements

Seeds of control and transgenic lines were germinated either on ½ MS agar medium or medium that also contained mannitol (150 mM) or NaCl (100 mM) at 22 °C and 16 h/8 h light-dark cycles. Plants were grown vertically on Petri dishes for 15 days and root lengths measured manually.

### Drought treatment in transgenic cotton

To study the drought response of transgenic *GhNAC2* expressing cotton plants, progeny of lines L-24 and L-30 (six plants each) were grown in pots in Neo peat (processed superior Coco peat) for two months under glasshouse conditions at 30 ± 2 °C. Watering was stopped after two months for a period of 15 days followed by rewatering and changes monitored. The number and percentage of leaves that abscised in response to drought was measured in each plant. For leaves that did not abscise but only showed wilting, the leaf angle between the petiole and the leaf laminar plane was measured (~10 leaves/plant). Root length measurements were carried out from the watered as well as drought treated plants after maturity (5 months 10 days).

### Physiological studies

For each experiment, homozygous progeny of the transgenic Arabidopsis lines 1, 4 and 8 (5–6 plants/line) were grown in pots in the culture room under white light and used. Gas exchange parameters were determined on 25-day old plants enclosed in a leaf chamber (3010-A) using the GFS-3000 instrument (Walz, Germany) attached with a fluorescence module (PAM-fluorometer 3055-FL, Walz, Germany). Steady-state photosynthesis rate (A), stomatal conductance (g_s_), transpiration rate (E) etc were measured at 70% humidity, 25 °C leaf temperature, 400 μmol mol^−1^ CO_2_ and PPFD adjusted to 400 μmol photons m^−2^ s^−1^. Intrinsic WUE was calculated as the A:E ratio.

### Relative water content and leaf drying assay

For leaf drying studies, Arabidopsis leaves (7^th^ leaf from bottom; 5 plants/line) from 25 day old plants were detached and allowed to dry at 27 °C. Leaf wilting and the gradual upturning of petioles were monitored. For relative water content, leaves of the homozygous transgenic and control lines were detached and changes in fresh weight monitored using an electronic balance. The tissues were rehydrated in water for 2 h until fully turgid, surface-dried, reweighed [turgid weight, TW] followed by oven drying at 80 °C for 48 h [dry weight, DW]. The relative water content (RWC) was calculated as:


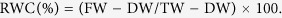


### Micro array analysis of transgenic lines

Total RNA from 20–22 day-old plants of wild-type (Col) and the *GhNAC2* over-expressing line, Line L-1, (three independent lots each), grown on ½MS containing soilrite, was extracted as described before. Affymetrix Gene Chip® ATH GeneChips (GEO accession number GSE8257) were used for microarray. Target preparation, hybridization, washing, staining and scanning were carried out as per instructions (Affymetrix, USA). Image files from hybridization data were analyzed to generate the probe intensity (.cel) files using the default settings of Affymetrix Gene Chip Operating Software (version 1.3). Normalization was carried out using GCRMA (GeneChip robust multiarray), PLIER (probe logarithmic intensity error) and RMA (robust multiarray average) using the ArrayAssist software (Stratagene, La Jolla, USA) and DNA-Chip analyser software (Li and Wong, 2001). Common probe sets in all the three analyses were chosen to identify differentially expressed genes that were statistically significant at p < 0.05 in the t tests and had a 2 fold or greater change between experimental samples.

### Statistical Analysis

The significance of correlations was tested by using linear regression, with *P* values of <0.05 considered statistically significant. Means were compared by using one-way analysis of variance and post hoc means comparison (Scheffé Test). Statistical analysis of plant growth phenotypes was performed using Microsoft Excel. Analysis of variance was used to compare the statistical difference based on Student’s t-test (n = 5–10).

## Additional Information

**How to cite this article**: Gunapati, S. *et al.* Expression of *GhNAC2* from *G. herbaceum*, improves root growth and imparts tolerance to drought in transgenic cotton and Arabidopsis. *Sci. Rep.* **6**, 24978; doi: 10.1038/srep24978 (2016).

## Supplementary Material

Supplementary Information

## Figures and Tables

**Table 1:** Physiological analysis of transgenic *GhNAC2* expressing Arabidopsis lines (L-1, L-4 and L-8).

Parameter/Line	Control	L-1	L-4	L-8
E (mmol H_2_O m^−2 ^s^−1^)	3.92 ± 0.90	2.01 ± 0.48**	1.98 ± 0.21**	2.51 ± 0.17*
A (μmol CO_2_ m^−2 ^s^−1^)	9.078 ± 1.98	5.63 ± 1.28*	4.53 ± 0.32**	5.40 ± 0.35*
Gs (mmol m^−2 ^s^−1^)	683 ± 229	330 ± 71*	433 ± 84	565 ± 119
A/E (μmol CO_2 _mmol H_2_O^−1^)	2.36 ± 0.52	2.83 ± 0.39	2.29 ± 0.16	2.17 ± 0.30

Gas exchange parameters were determined on plants enclosed in a leaf chamber (3010-A) using the GFS-3000 instrument as described in methods. Vertical bars indicate ± SD from five plants of each independent line. Parameters in bold represent parameters that are significantly different at *P < 0.05, **P < 0.01.

E = Transpiration; A = photosynthetic rate; Gs = conductance; A/E = water use efficiency.

**Table 2:** Microarray analysis showing differentially regulated hormone pathway and stress related genes in transgenic *GhNAC2* expressing Arabidopsis line L-1 compared to Col.

Gene	Nature of Regulation	Status	Fold change
Abscisic acid			
AtPYL8	Positive (ABA receptor)	Up	2.79
CYP707A3	Negative (reduction of ABA levels)	Down	4.48
AHG3	Negative (encodes protein phosphatase that inhibits SnRKs)	Down	2.66
AtWRKY18	Negative	Down	9.6
AtWRKY40	Negative	Down	11.64
AtWRKY70	Negative regulator of stomatal closure	Down	3.73
CIPK14	Negative regulator of ABA responses	Down	3.0
Ethylene			
ACS6	Positive (Ethylene biosynthesis)	Down	8.6
MPK3	Positive (MAPK-activator of stress ethylene)	Down	2.46
MKK9	Positive (activator of MAPK3/6)	Down	2.3
ERF1	Positive (Ethylene response)	Down	5.2
ERF2	Positive (Ethylene response)	Down	2.74
ERF6	Positive (Ethylene response)	Down	5
ERF11	Ethylene/ABA response	Down	11.5
EBF2	Negative (Depletion of EIN3)	Up	3.1
WRKY33	Positive (Activator of ERF6)	Down	4.44
Jasmonic acid			
LOX3	Positive (JA biosynthesis)	Down	13.6
LOX4		Down	53.3
AOS		Down	2.29
AOC3		Down	22
ERD12		Down	3
CYP94C1	Negative (JA catabolism)	Down	11.31
JAZ1	Negative (JA response inhibitors)	Down	9.86
JAZ2		Down	3.3
JAZ5		Down	19
JAZ6		Down	3
JAZ7		Down	50.7
JAZ9		Down	2.89
MYC2	Positive (TF that activates JA response)	Down	6.5
Gibberellic acid			
GA2OX2	Negative (Depletion of active GA)	Down	2.72
GASA1	Positive (GA responsive)	Up	4.11
Stress responsive genes (that reduce growth upon over-expression)			
RD26	Positive (Drought response)	Down	6.6
NAC3	Positive (ANAC055; drought responsive)	Down	4.47
RD29A	Positive (Drought response)	Down	4.87
MYB44	Negative regulator of stomatal closure (Activator of WRKY70)	Down	5.38
ZAT10/STZ	positive (Stress response)	Down	7.7
ERD9	positive (Glutathione S Transferase)	Down	4.1
ERD5	Positive (Proline dehydrogenase)	Up	12
