# Corrigendum: Expression of *GhNAC2* from *G. herbaceum*, improves root growth and imparts tolerance to drought in transgenic cotton and Arabidopsis

**DOI:** 10.1038/srep34897

**Published:** 2016-10-10

**Authors:** Samatha Gunapati, Ram Naresh, Sanjay Ranjan, Deepti Nigam, Aradhana Hans, Praveen C. Verma, Rekha Gadre, Uday V. Pathre, Aniruddha P. Sane, Vidhu A. Sane

Scientific Reports 6: Article number: 24978; 10.1038/srep24978 published online: 04 26 2016; updated: 10 10 2016.

This Article contains errors in Reference 44 which was incorrectly given as:

‘Kumar, M., Singh, H., Shukla, A., Verma, P. C. & Singh, P. Induction and establishment of somatic embryogenesis in elite Indian cotton cultivar (Gossypium hirsutum L. cv Khandwa-2). *Plant Signal. Behav.* **8(10)**, e26762 (2013)’.

The correct reference is listed below:

Kumar, M., Shukla, A. K., Singh, H., Verma, P. C. & Singh, P. K. A genotype-independent Agrobacterium mediated transformation of germinated embryo of cotton (Gossypium hirsutum L.). *International Journal of Bio-Technology and Research* **3(1)**, 81–90 (2013).

