# Supplementary material for: Expression of GhNAC2 from G. herbaceum, improves root growth and imparts tolerance to drought in transgenic cotton and Arabidopsis
Source: Sci Rep. 2016 Apr 26;6:24978. doi: 10.1038/srep24978 (PMC4844984; doi:10.1038/srep24978)
Supplement: Supplementary Information [file srep24978-s1.pdf]

Expression of *GhNAC2* from *G. herbaceum*, improves root growth and imparts tolerance to drought in transgenic cotton and Arabidopsis

Samatha Gunapati[1], Ram naresh[1], Sanjay Ranjan[2], Deepti Nigam[3], Aradhana Hans[4], Praveen C. Verma[4], Rekha Gadre[5], Uday V. Pathre[2], Aniruddha P. Sane[1], Vidhu A. Sane[1]*

[1]Plant Gene Expression Lab, [2]Dept. of Plant Physiology, [3]Dept. of Bioinformatics, [4]Plant tissue culture, CSIR- National Botanical Research Institute, Lucknow-226001, India.

[5]Dept of Biochemistry, DeviAhilyaBai University, Indore-452001, India

* Corresponding author. Tel : 91 522 2297978; Fax: 91 522 2205836

E.mail: sanevidhu@rediffmail.com, va.sane@nbri.res.in

Running title: *GhNAC2* improves growth and drought tolerance

Supplementary figures & tables

Supplementary Table S1. Sequences of primers used for various studies

Primer name	Sequence
GhNAC (F-1)	5’CCACCTAGAGCGAGTGTGA3’
GhNACF0	5’ GTTGGATCCATGACAGCATCGGAG 3’
GhNACRP	5’CAGGATCCGTCCTGGAGCGGGGACAG3’
GhNACR0	5’GTGGATCCCTAAAATGGCTTCTGCAGG3’
GhNACF3	5’ ATGTGCATCGCAGTCCATC 3’
GhNACF4	5’ TCCTTGGGACCTTCCAGATT 3’
GhNACR3	5’ CTCCGTACAACGCCAAATCT 3’
GhNACF1	5’ GGATTYMGRTTCCATCCHAC 3’
GhNACF2	5’ THTTTRYAARTDYGAKCCTTGG 3’
GhNACR1	5’ CGRTAYTCRTGCATAAYCC 3’
GhNACR2	5’ TTRTCNKYTCCSGTHGCYTTCCA 3’
GhActqF1	5’TGGCACTCGACTTCGAGCAGGA 3’
GhActqR1	5’CAGGACAACGGAAACGCTCTGC 3
3’AP	5’GGCCACGCGTCGACTAGTAC(T)17 3’

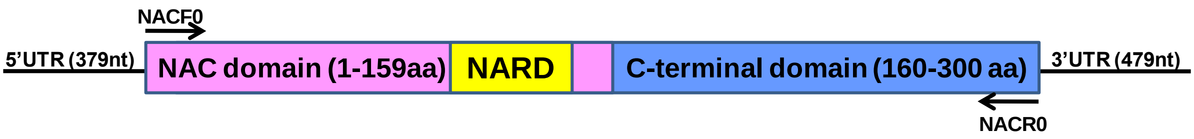

Supplementary Figure S1

Figure S1. Schematic representation of the GhNAC2 coding and flanking portions. The NAC domain and the repressor NARD motif within are shown as boxes. Arrows mark the positions of the primers used for amplification of full-length *GhNAC2* and *GhΔNAC2*.

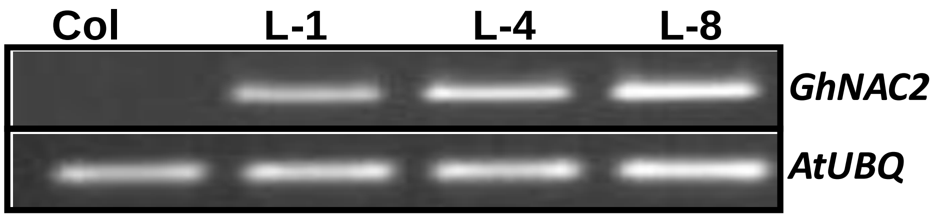

Supplementary Figure S2

Figure S2. Transcript accumulation of *GhNAC2* in leaves of independent transgenic Arabidopsis lines.

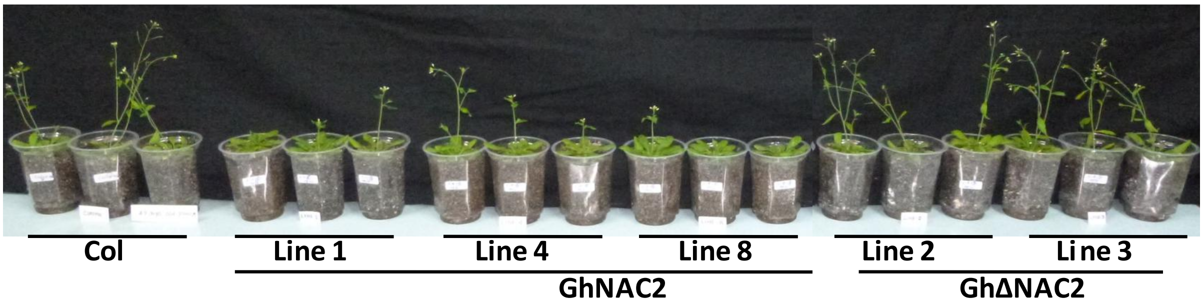

Supplementary Figure S3

Figure S3. Differences in bolting of control and transgenic *GhNAC2* and *GhΔNAC2* expressing Arabidopsis lines.

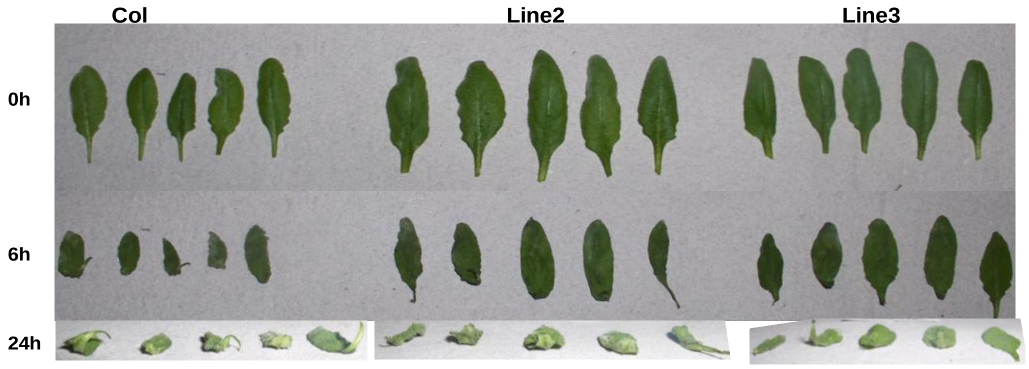

Supplementary Figure S4

Figure S4. Comparative analysis of leaf drying in excised leaves of control and transgenic *GhΔNAC2* expressing plants.

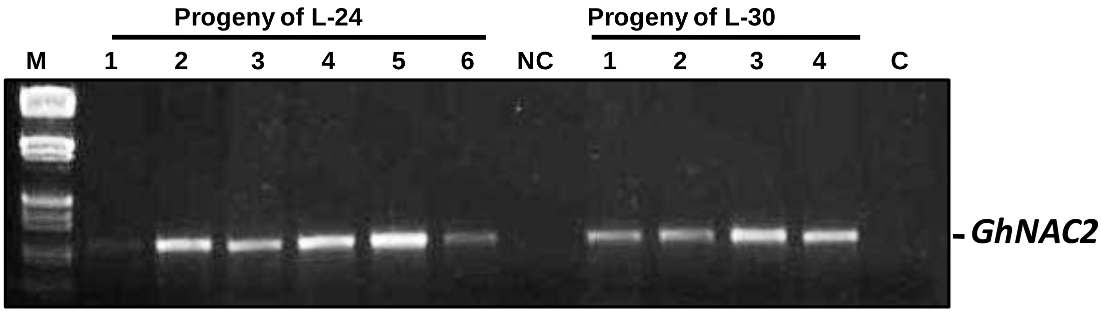

Supplementary Figure S5

Figure S5. PCR analysis showing the presence of *GhNAC2* in transgenic cotton lines (progeny of L-24 and L-30). Primers specific for CaMV35S promoter (forward) and GUS (reverse) were used for amplification of the intervening region containing *GhNAC2*
